# Long-Acting Injectable Statins—Is It Time for a Paradigm Shift?

**DOI:** 10.3390/molecules24152685

**Published:** 2019-07-24

**Authors:** Lee M. Tatham, Neill J. Liptrott, Steve P. Rannard, Andrew Owen

**Affiliations:** 1 Department of Molecular and Clinical Pharmacology, Materials Innovation Factory, University of Liverpool, Liverpool L7 3NY, UK; 2 Tandem Nano Ltd., Liverpool L22 3GL, UK; 3 Department of Chemistry, Materials Innovation Factory, University of Liverpool, Liverpool L7 3NY, UK

**Keywords:** nanoparticles, depot, sustained release, adherence

## Abstract

In recent years, advances in pharmaceutical processing technologies have resulted in development of medicines that provide therapeutic pharmacokinetic exposure for a period ranging from weeks to months following a single parenteral administration. Benefits for adherence, dose and patient satisfaction have been witnessed across a range of indications from contraception to schizophrenia, with a range of long-acting medicines also in development for infectious diseases such as HIV. Existing drugs that have successfully been formulated as long-acting injectable formulations have long pharmacokinetic half-lives, low target plasma exposures, and low aqueous solubility. Of the statins that are clinically used currently, atorvastatin, rosuvastatin, and pitavastatin may have compatibility with this approach. The case for development of long-acting injectable statins is set out within this manuscript for this important class of life-saving drugs. An overview of some of the potential development and implementation challenges is also presented.

Cardiovascular disease remains the leading cause of death in older adults, accounting for 60% of deaths in those aged 85 years or older [1,2]. Hypercholesterolemia is a major risk factor for the progression of atherosclerosis, the primary cause of coronary heart disease [3]. Statins are effective in lowering blood cholesterol and are widely used in the pharmaceutical prevention of cardiovascular disease [4]. It is estimated that with each 1.0 mmol/L reduction in blood low density lipoprotein (LDL) cholesterol, there is a corresponding 22% reduction in the rate of major vascular events (defined as coronary death, non-fatal myocardial infraction, coronary revascularisation or stroke) and a 14% reduction in overall vascular mortality [5,6]. Statins are the most commonly prescribed lipid-modifying therapies, with over 25 million people worldwide receiving therapy [7]. Additionally, the use of statins in those aged 79 years and older has increased four-fold in proportion to the rise in prevalence of cardiovascular disease for this population in the past decade [1,8].

Seven statins are currently approved for clinical use in at least one country [9] (Table 1). Despite differences in their chemistry and pharmacokinetics, all statins lower blood cholesterol through the competitive inhibition of the enzyme, 3-hydroxy-3-methylglutaryl-coenzyme A (HMG-CoA) reductase, which catalyses the conversion of HMG-CoA to mevalonate, the rate limiting step in cholesterol synthesis. The competitive inhibition of HMG-CoA decreases hepatocyte cholesterol synthesis and the reduced intracellular cholesterol concentration induces the expression of LDL receptors on the hepatocyte cell surface. This results in increased extraction of LDL cholesterol from the blood and effectively reduces circulating LDL cholesterol [4,10]. Statins have also been shown to have beneficial effects on other lipid parameters including increasing high-density lipoprotein (HDL) cholesterol and decreasing triglyceride concentrations in the blood [11]. Additionally, lipoprotein abnormalities are frequently observed in patients with metabolic syndrome or type 2 diabetes [12]. A significant number of these patients remain at risk of cardiovascular disease despite achieving the recommended LDL cholesterol concentration [13]. Fibrates are commonly co-administered in patients with high residual risk and, like statins, are lipid profile modifying drugs [14]. Fibrates regulate the expression of genes involved in multiple metabolic pathways leading to reductions in plasma triglyceride concentrations and increasing HDL cholesterol levels [15]. Therapeutic interventions based on statin and fibrate combinational therapy has been shown to be particularly effective in patients when a number of lipid parameters need to be addressed and is not achievable with statin therapy alone [14].

Despite the clinical utility of statins, there are limitations associated with their current use. Of particular relevance to the current manuscript, a recent systematic review highlighted that rates of patient adherence to statins ranged between 17.8% and 79.2% [16], and a previous meta-analysis showed that only 60% of 1,978,919 patients were adherent to statins being used for primary or secondary treatment. Importantly, adherence is related to the efficacy of statins. Observational studies have shown a relationship of adherence with cholesterol and cardiovascular disease, with patients having adherence ≥90% seemingly more likely to have a ≥25% reduction in LDL than those in the 80–89% adherence range [16,17,18]. Older age is well known to be associated with lower adherence to medication generally, and retirement has been shown to specifically impact adherence to statins [19]. Accordingly, the need for improved strategies and interventions to tackle non-adherence to statin therapy has been recognised [20].

In recent years, long-acting drug delivery has emerged as an effective approach for management (therapy and prevention) of indications requiring chronic drug administration, particularly where low patient adherence to medication negatively impacts outcomes. Long-acting terminology is already applied to statins to discriminate long half-life from short half-life drugs and so definition warrants clarification. Accordingly, long-acting in the context of this manuscript is defined as the advanced drug delivery approaches that provide therapeutic drug exposure for a period ranging from several weeks to several months after a single dose. Several such approaches to long-acting delivery exist, each presenting different development, financial, clinical, and logistical challenges and opportunities. Drug delivery implants have proven highly successful for potent contraceptive agents that require low target plasma concentrations. However, when higher plasma concentrations are needed, insufficient drug can be loaded into tolerably sized implants, and multiple implants are frequently used to obtain sufficient and sustained drug exposure even for highly potent contraceptives. Conversely, long-acting injectable (LAI) medicines are widely applicable across indications providing drug exposure for a period between two and 12 weeks from a single parenteral administration (intramuscular or subcutaneous). Licensed LAIs are available for contraception [21], schizophrenia [22,23], opioid addiction [24], androgen ablation [25], and hypogonadism [26]. LAI medicines are also in late stage development for the treatment of HIV [27], with preclinical proof-of-concept emerging across infectious diseases [28,29,30].

As well as the obvious advantages of tackling issues of non-adherence, LAI medicines have proven highly popular with patients with schizophrenia across many country contexts [31,32]. Early signs of popularity are also evident for the long-acting HIV medicines in phase II trials and an appetite for the approach from general patient groups [27,33]. Many patients become tired of taking oral pills on a daily basis, which is also a constant reminder of their disease that is mitigated when moving from oral to long-acting therapy.

Existing LAI rely upon oil depot formulations (e.g., fluphenazine, testosterone), polymer microsphere encapsulation (e.g., naltrexone, risperidone), or aqueous particle suspensions (e.g., paliperidone palmitate, olanzapine pamoate, leuprolide mesylate, aripiprazole lauroxil, medroxyprogesterone acetate, rilpivirine, cabotegravir). Polymer microspheres require high polymer to drug ratios to control drug release, resulting in low active drug ratio and restricting the achievable duration when high doses are required. Oil depot formulations have needed new prodrugs to increase lipophilicity and achieve high oil concentrations to provide adequate doses (e.g., haloperidol decanoate, fluphenazine decanoate, testosterone undecanoate). They must also be administered slowly and are commonly associated with administration pain and skin reactions that can persist for up to three months. Aqueous particle suspensions are more tolerable, some require prodrug derivitisation but existing orally administered drugs are sometimes compatible directly, which reduces the regulatory bar for rapid introduction. Importantly, despite the need for advanced processing technologies that sometimes manipulate the drug at the nanometer scale, many of these approaches are highly cost effective with emerging interest for applications even in low- and middle-income countries [34].

There is currently a paucity of knowledge relating to the mechanisms for drug absorption and what makes the ideal LAI drug molecule [35]. However, existing LAI medicines have a long pharmacokinetic half-life, low water solubility, and low target plasma concentrations. Oral half-life correlates with LAI half-life but is extended through flip-flop kinetics. In a basic sense, a drug cannot be cleared until it is absorbed so the rate of drug elimination approaches the rate of absorption. Water-soluble drugs cannot be utlilised because of rapid dissolution and potential for dose-dumping. Low target concentrations are a prerequisite for the approach because high plasma concentrations are harder to achieve and sustain for long periods of time.

A review of the data for existing clinically used statins reveals that three exhibit physicochemical and pharmacological properties that may make them compatible with formation of aqueous particle suspensions for LAI delivery. The oral pharmacokinetic half-life, aqueous solubility, and plasma concentration achieved after oral administration (surrogate for plasma target concentration) are summarised in Table 1. Atorvastatin, rosuvastatin, and pitavastatin have low aqueous solubility, low target plasma concentrations, and reasonably long half-lives, all broadly within the range of drugs that have previously been successfully developed into LAI formulations.

When administered orally, absorption of statins is rapid but highly variable (Table 1) [36,37,38,39]. Most existing LAI medicines exhibit greater pharmacokinetic variability than their oral counterpart medicines and careful monitoring may be needed for an LAI equivalent to establish effective and safe exposures. Absorption is also affected by time-of-day administration and statins with short half-lives of 3 h or less are best administered in the evening when the rate of cholesterol synthesis is highest [4]. Statins with longer half-lives such as atorvastatin can be administered any time of day and shows greater efficacy for lowering LDL cholesterol compared to older statins, partly due to the longer residence time of the drug [40]. Clearly, the need to consider time of day dosing would be obviated for an LAI because administration would occur monthly or less frequently with a flattening of the pharmacokinetic profile across the dosing interval.

Plasma protein binding is extensive for all the statins except pravastatin, resulting in low systemic exposure to the unbound pharmacologically active drug (Table 1) [36]. Statins are amphiphilic and enter hepatocytes either directly by membrane interactions in the case of lipophilic agents or via carrier proteins in the case of hydrophilic agents [41]. Additionally, the uptake of statins is influenced by the drug transporters OATP1B1 and/or ABCC2 in the liver [42]. Most statins undergo first-pass liver metabolism, significantly reducing systemic bioavailability [43]. Atorvastatin, simvastatin and lovastatin are primarily metabolised by CYP3A4 although other pathways play a minor role in metabolic processing [11]. Active metabolites have been described for these drugs, inhibiting HMG-CoA reductase. The main active metabolites for atorvastatin are 2-hydroxy- and 4-hydroxy-atorvastatin acid [44]. Fluvastatin is primarily metabolised by CYP2C9 although CYP3A4 and CYP2D6 may also play a modest role [45]. Pravastatin, pitavastatin and rosuvastatin do not undergo substantial metabolism by CYP isoenzymes [42]. The principal route of elimination for most of the statins is via biliary excretion following metabolism by the liver. Pravastatin and rosuvastatin are eliminated mostly as unchanged drug by both the liver and kidneys [4].

Simvastatin and lovastatin are administered orally as lactone prodrugs and are enzymatically hydrolysed in vivo to their active hydroxy-acid form. The remaining statins are administered in their active hydroxy-acid form [3,4]. It is estimated that equal amounts of the acid and lactone forms of atorvastatin and simvastatin are present in human plasma whereas less than 5% of fluvastatin and pravastatin is present in the lactone form [46,47,48]. It has been suggested that conversion from the acid to the lactone form is mediated by glucuronidation via uridine diphosphate (UDP)-glucuronosyltransferase 1A1 (UGT1A1) and UGT1A3 and/or β-oxidation reactions in the liver [49,50]. Lactonised statins can be metabolised via a number of pathways, including, directly by CYP isoenzymes which occurs more rapidly than for the corresponding acid forms, or the lactonised statin may be converted back to the acid form by hydrolysis via chemical interconversion by estereases/paraoxenases and subsequently be metabolised [42,48,51]. Delivery of drugs through formation of LAI medicines doesn’t alter the composition of the drug systemically, even with approaches involving nanotechnology. Unlike other nanotechnology-based approaches to drug delivery, aqueous particle dispersions are composed of only drugs with excipient that are already pharmaceutically accepted and chosen from the Food and Drug Administration (FDA) Center for Drug Evaluation and Research (CDER) list. Importantly therefore, the drug is in the same form within the systemic circulation following absorption from oral or LAI delivery, meaning that the distribution, metabolism, and elimination pathways remain unchanged. However, almost all LAI medicines require lower doses than their oral counterparts when averaged across the dosing interval. For example, rilpivirine and cabotegravir require 1400 mg and 1700 mg for 2 months of therapy when given orally, but 600 mg and 900 mg, respectively, exhibit comparable efficacy when given as LAI [27]. Similarly, reductions in dose are evident across other LAI medicines [35].

Statins are safe and effective in the majority of patients [7]. However, they are associated with muscle complaints including myalgia, cramps and weakness that can compromise quality of life and reduce compliance to medication [52,53]. The incidence of patient reported myalgia during statin therapy ranges from 1% in controlled studies to 25% in clinical reports [52,54]. The clinical spectrum of statin induced myopathy (SIM) ranges from asymptomatic elevations in creatine kinase (CK) without muscle pain to, in very rare incidences, potentially life-threatening rhabdomyolysis with high CK and potential for acute kidney injury [7]. Therefore, if statins are to be developed into LAI medicines then it will be critical to consider the implications for myopathy and use this to inform drug selection. The mechanisms that underpin SIM are not fully understood, although more lipophilic statins such as simvastatin and lovastatin appear more toxic [41,55]. A number of risk parameters have been reported for developing SIM including patient factors, such as advanced age, low BMI, comorbidities, excessive physical exercise, and personal or family history of SIM [7]. Additionally, the combination of statins with drugs that are known inhibitors of CYP3A4, CYP2C9 or OATP1B1 potentially increases the risk of SIM. In several studies, although not all, plasma concentrations of statin or statin metabolites were higher in patients with associated myopathy [7,56]. If myopathy is concentration-dependent, then there may be advantages to LAI-based delivery because high daily maximum plasma concentrations, required to maintain overall exposure, are avoided by the approach.

Many LAI medicines require intramuscular administration in order to tolerably accommodate the high doses required for long durations of exposure. For example, a 3 mL intramuscular injection every eight weeks is being progressed for each of the LAI antiretrovirals cabotegravir and rilpivirine [27]. This may seem counterintuitive for drugs that exhibit muscle toxicities. However, a number of studies have identified the involvement of metabolites in the development of SIM. Skottheim et al., incubated primary human myoblasts with increasing concentrations of lactone and acid forms of different statins. Quantitative measurements of myotoxicity revealed that the lactone forms of all the studied statins decreased the number of living myotubes compared to the respective acid forms. Atorvastatin-, fluvastatin-, pravastatin- and simvastatin-lactone showed a 14-, 26-, 23- and 37-fold increase in myotoxicity compared to the corresponding acid forms, respectively [48]. Hermann et al., compared the pharmacokinetics of atorvastatin and its metabolites in healthy control patients and patients with SIM. The patients with myopathy had a 2.4- and 3.1-fold increase in exposure in atorvastatin-lactone and para-hydroxy-atorvastatin, respectively. However, no significant difference in exposure to the pharmacologically active acid form was observed between the two groups. The authors noted that increased exposure to atorvastatin-lactone was observed in patients with and without concomitant treatment with CYP3A4 inhibitors. Additionally, atorvastatin-lactone is significantly more lipophilic than the acid form and expected to have a higher tissue penetration. Therefore, patients with increased exposure to atorvastatin-lactone, either inherently or due to interacting drugs, could have increased tissue exposure which may account for the increased risk for myopathy [57]. More recently, it has been demonstrated that lactonisation of atorvastatin is mainly mediated by UGT1A3, with an increased rate in carriers of the UGT1A3*2 haplotype. This suggests that lactonisation is at least partly enzymatically controlled [58]. If the causative metabolite for SIM is generated predominantly within the liver, then the route of administration may not influence the incidence of this toxicity. The avoidance of oral first-pass metabolism by parenteral administration may also prove beneficial, but this would need to be empirically assessed as part of any LAI statin development programme.

It is important to recognise that patients with hyperlipidemia undergoing therapy with statins frequently also require treatment for comorbid conditions such as diabetes and cardiovascular disease. This clearly has important implications if LAI statins are to be effectively developed and deployed. Firstly, patients that are non-adherent to statin therapy are also more likely to also be non-adherent to their co-medications. Clearly, availability of LAI statins will not mitigate the need for strict adherence to the co-medications and alternative strategies may also be needed for these other agents. Secondly, there is currently limited knowledge regarding whether the drug–drug interaction potential is altered when drugs are formulated for LAI. It is possible that for some interactions, the avoidance of the gastrointestinal tract and first-pass metabolism may decrease the likelihood or magnitude. However, further work is clearly needed to understand the liability of LAIs as both perpetrators and victims of drug interactions.

In summary, benefits for adherence, dose requirement and patient satisfaction have been achieved across indications by the development of LAI medicines. Three existing statins may be compatible with LAI development using advances in nanoparticle formulation technologies. Non-adherence to statins has important consequences for therapy outcome that may be avoided by LAI deployment. The approach may not necessarily exacerbate issues with toxicity of statins, and may be mitigated by the requirement for lower doses, lower maximum plasma exposures and careful analysis relating to route of administration. Benefits must be weighed against the potential for higher variability in drug exposure after LAI administration. However, development of LAI statin medications seems worthy of further consideration.

## Figures and Tables

**Table 1:** Relevant pharmacokinetic, pharmacodynamic and physiochemical properties of statins.

	Standard Daily Dose (mg)	Oral Bioavailability (%)	IC_50_ HMG-CoA Reductase (nM)	Aqueous Solubility (mg/mL)	Plasma Target Concentration (ng/mL) *	Elimination Half-Life (h)
Pravastatin	10–40 [43]	18 [43]	4 [43]	0.006 [59]	~1.0 ^a^ [60]	1.8 [4]
Lovastatin	10–40 [43]	5 [43]	2–4 [43]	0.0004 [59]	~1.7 ^a^ [39]	3 [4]
Simvastatin	10–40 [43]	<5 [43]	1–2 [43]	0.01 [59]	1.4 ^b^ [61]	2 [4]
Fluvastatin	80 [43]	30 [43]	3–10 [43]	0.0005 [59]	1.9 ^b^ [62]	1.2 [4]
Atorvastatin	10–80 [43]	12 [43]	1.16 [43]	0.000001 [59]	5.0 ^b^ [63]	14 [4]
Rosuvastatin	5–40 [43]	20 [43]	0.16 [43]	0.02 [59]	0.5 ^b^ [64]	19 [4]
Pitavastatin	1–4 [43]	60 [43]	0.1 [43]	4 × 10^−4^ [59]	2.5 ^b^ [65]	11 [4]

* Steady-state C_trough_ at 12 h ^a^ or 24 h ^b^ post dose.
